# CAF derived IL-33 mediated EMT to promote the metastasis of LSCC cells

**DOI:** 10.1186/s40001-025-03431-4

**Published:** 2025-11-21

**Authors:** Liyun Yang, Jieyu Zhou, Shiyan Chen, Yanmei Wang, Shuixian Huang

**Affiliations:** 1 Department of Otolaryngology, Shanghai Pudong New Area Gongli Hospital, Shanghai, 200135 China; 2 Department of Otolaryngology-Head and Neck Surgery, Shanghai Ninth People’s Hospital, Shanghai Jiaotong University School of Medicine, Shanghai, 200011, China; 3 Ear Institute, Shanghai Jiaotong University School of Medicine, Shanghai, 200011 China; 4 The Hospital of the 94303 Troop of the Peoples Liberation Army, Weifang, 261000 China; 5 Department of Otolaryngology, Qingdao Central Hospital‚ University of Health and Rehabilitation Sciences (Qingdao Central Hospital), Qingdao, 266000 China

**Keywords:** EMT, LSCC, CAF, IL-33, Metastasis

## Abstract

**Background:**

The metastasis of laryngeal squamous cell carcinoma (LSCC) is not only caused by the tumor itself but is also closely related to cancer-associated fibroblasts (CAFs). The epithelial-mesenchymal transition (EMT) serves as a key event during its metastasis. However, the specific mechanisms underlying LSCC metastasis remain uncertain.

**Methods:**

A wound healing assay was utilized to evaluate the migratory capacity of LSCC cells (TU686 and TU212 cells). Immunofluorescence staining and Western blot analysis were conducted to demonstrate the expression levels of associated proteins. Migration and invasion assays were employed to assess the migration and invasion abilities of LSCC cells in vitro. A nude mouse metastasis model was used to detect LSCC metastasis in vivo.

**Results:**

Our results revealed that interleukin-33 (IL-33) enhanced the migratory, invasive, and EMT capabilities of LSCC cells. In the co-culture model of LSCC cells and CAFs, silencing the expression of IL-33 inhibited the migratory, invasive, and metastasis potential of LSCC cells both in vitro and in vivo.

**Conclusion:**

IL-33 derived from CAFs mediates EMT to promote the metastasis of LSCC cells. The findings of our study not only provide a new mechanism for the activation of CAFs and the metastasis of LSCC but also offer theoretical significance and application value for more effective prevention and treatment strategies in clinical practice.

**Supplementary Information:**

The online version contains supplementary material available at 10.1186/s40001-025-03431-4.

## Introduction

Laryngeal squamous cell carcinoma (LSCC) is one of the common malignant tumors in the head and neck region [1]. The primary clinical manifestation of laryngeal cancer is hoarseness. Although the primary treatment for LSCC patients is surgical intervention, their prognosis remains poor, mainly due to the metastasis of LSCC. Therefore, it is crucial to elucidate the specific molecular mechanisms involved and identify new therapeutic targets to develop more effective prevention and treatment strategies for clinical practice.

Epithelial-mesenchymal transition (EMT) is a process where epithelial cells lose their intercellular connections and polarity, transforming into mesenchymal cells, a phenomenon widely observed in cancer metastasis [2]. The metastasis of LSCC is closely associated with EMT [3, 4]. Understanding EMT is vital as it relates to the biological characteristics of tumors, potentially providing more effective prevention and treatment strategies for tumor metastasis. However, the molecular basis and regulatory mechanisms of EMT in LSCC require further investigation. A deeper understanding of the molecular mechanism of EMT in LSCC will not only aid in comprehending the specific mechanisms of LSCC metastasis but also offer more effective prevention and treatment strategies for clinical practice, thereby holding theoretical significance and application value.

It is known that EMT, as a key event in tumor cell migration, invasion, and metastasis, is linked to strong interactions between the cancer microenvironment and cancer cells [5]. Cancer-associated fibroblasts (CAFs) are a diverse group of cells originating from various sources, including mesenchymal fibroblasts, vascular beds, epithelial tumor cells, normal epithelial cells, and bone marrow mesenchymal stem cells. Fibroblasts are the primary cell type within the stromal composition and participate in the dynamic equilibrium of tissue structure and function. They play a significant role in tumor progression, such as contributing to the synthesis, deposition, and remodeling of the tumor matrix, serving as a major source of paracrine cytokines [6, 7]. Functionally, normal fibroblasts can suppress the malignant phenotype of epithelial cells and maintain epithelial stability. Conversely, CAFs promote the malignant transformation and growth of epithelial cells by remodeling the tumor matrix and secreting inflammatory factors like TGF-β and IGF [8]. Therefore, in-depth study of CAFs is essential for clarifying the mechanisms of tumorigenesis and progression and provides new theoretical foundations and intervention strategies for the early diagnosis of tumors. Hence, the activation of CAFs is critical for EMT in LSCC.

In our research, the results indicated that interleukin-33 (IL-33) accelerates the migratory, invasive, and EMT capabilities of LSCC cells. In the co-culture model of LSCC cells and CAFs, silencing the expression of IL-33 inhibited the migratory, invasive, and metastasis potential of LSCC cells both in vitro and in vivo. To summarize, IL-33 derived from CAFs mediates EMT to promote the metastasis of LSCC cells. The findings of this study not only provide a new mechanism for the activation of CAFs and the metastasis of LSCC but also offer theoretical significance and application value for more effective prevention and treatment strategies in clinical practice.

## Results

### IL-33 accelerated the mobility, migration, and invasion of LSCC cells

**Fig. 1:**
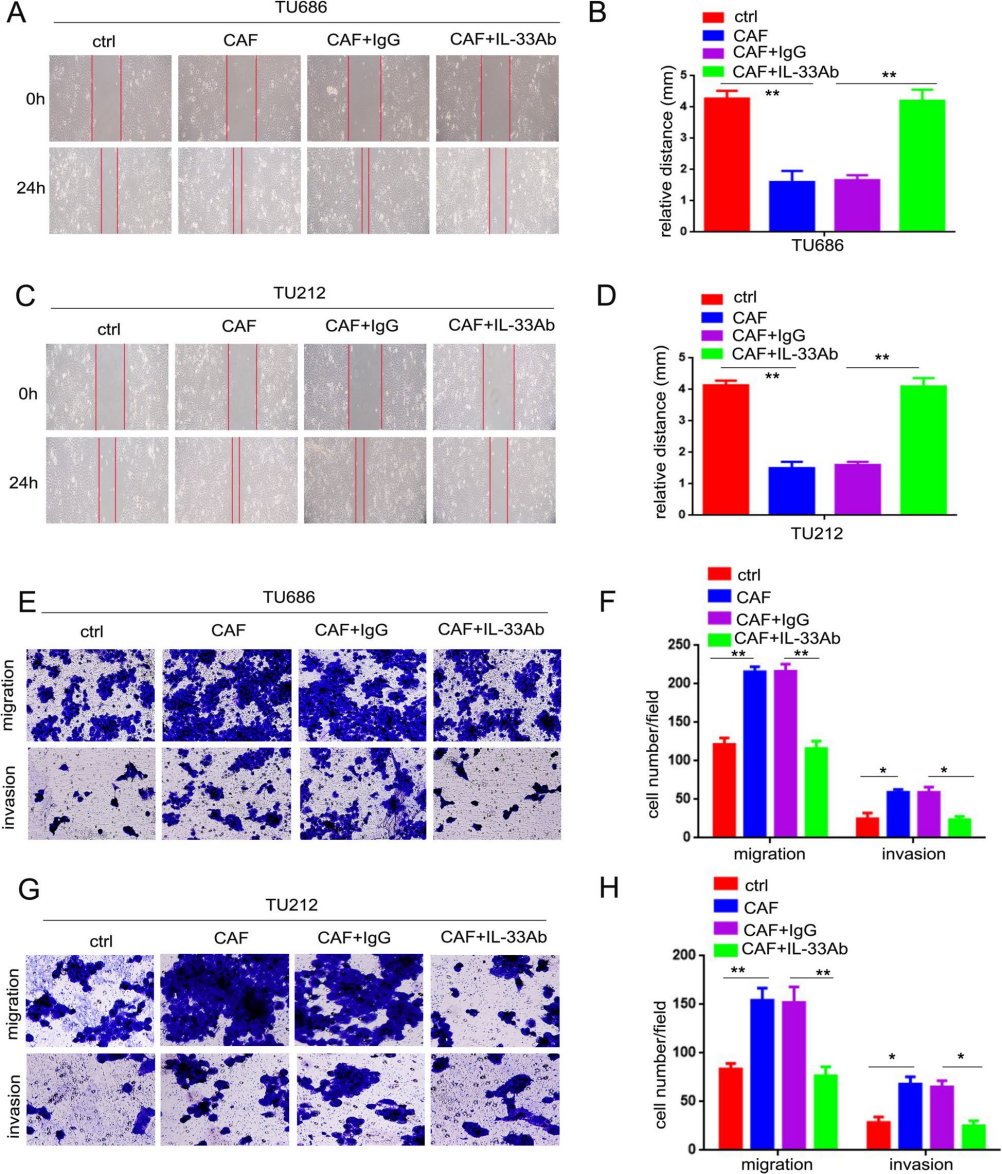
IL-33 played an important role in promoting the movement, migration, and invasion of LSCC cells. **A–C** The mobility of TU686 and TU212 cells was analyzed via a wound-healing assay. **B–D** The relative distance of TU686 and TU212 cells (**p < 0.01). **E–G** The migration and invasion abilities of TU686 and TU212 cells were analyzed by Transwell assay. **F–H** The cell number in each field of TU686 and TU212 cells (*p < 0.05, **p < 0.01)

During the process of carcinogenesis, inflammatory factors can promote tumor metastasis through multiple molecular mechanisms [9]. To further investigate this, the mobility, migration, and invasion abilities of LSCC cells were assessed by blocking IL-33 stimulation using a neutralizing antibody. As shown in Fig. 1A, C, TU212 and TU686 cells co-cultured with CAFs (designated as TU686/CAF and TU212/CAF cells) demonstrated enhanced mobility compared to TU686 and TU212 cells cultured alone. When a neutralizing IL-33 antibody was added to the co-culture system to block IL-33, the mobility of TU686 and TU212 cells was significantly reduced, with statistical significance (Fig. 1B, D, p < 0.05). Moreover, TU686/CAF (215 ± 6.6) and TU212/CAF cells (154 ± 12.5) exhibited enhanced migration compared to TU686 (121 ± 8.5) and TU212 cells (83 ± 5.5, Fig. 1E–H, p < 0.01) cultured alone. Similarly, when the neutralizing IL-33 antibody was added to the co-culture system, the migration of TU686 and TU212 cells co-cultured with CAFs was significantly reduced by the anti-IL-33 neutralizing antibody (TU686 cells: neutralizing IL-33 group, 116 ± 9.6 cells per field; isotype control group, 216 ± 9.4 cells per field. TU212 cells: neutralizing IL-33 group, 76 ± 9.1 cells per field; isotype control group, 152 ± 16.1 cells per field, Fig. 1E–H, p < 0.01). Additionally, TU686/CAF (59 ± 3.5) and TU212/CAF cells (68 ± 7.6) showed enhanced invasiveness compared to TU686 (24 ± 7.5) and TU212 cells (28 ± 5.5, Fig. 1E–H, p < 0.05) cultured alone. The invasive ability of TU686 and TU212 cells co-cultured with CAFs was also significantly reduced by the addition of the anti-IL-33 neutralizing antibody (TU686 cells: neutralizing IL-33 group, 23 ± 4.5 cells; isotype control group, 59 ± 6.6 cells. TU212 cells: neutralizing IL-33 group, 25 ± 5.3 cells; isotype control group, 65 ± 6.6 cells, Fig. 1E–H, p < 0.05). These results indicate that IL-33 accelerated the mobility, migration, and invasion of LSCC cells.

### IL-33 accelerated the EMT of LSCC cells

**Fig. 2:**
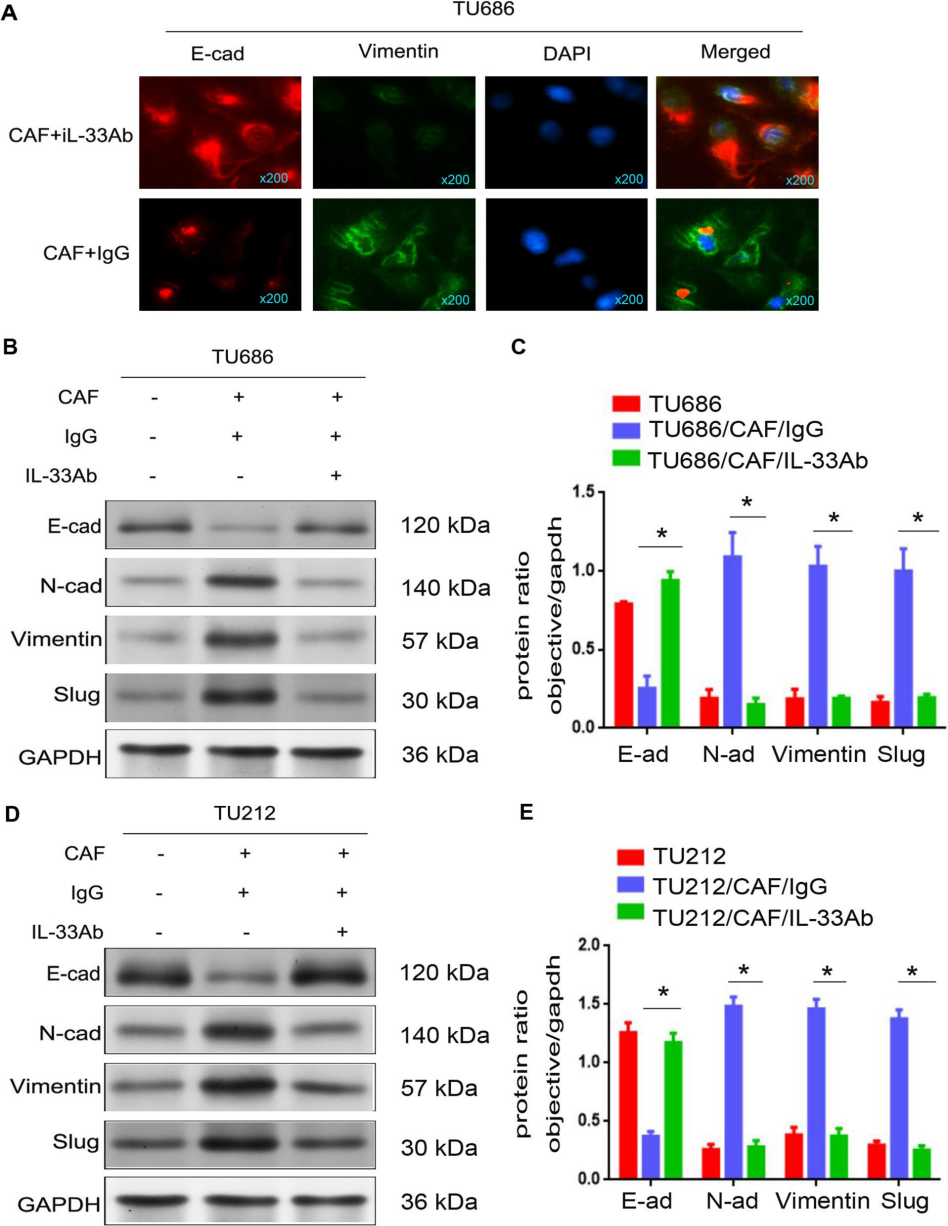
IL-33 promoted the EMT of LSCC cells. **A** The expressions of E-cad and Vimentin were detected by immunofluorescence assay. **B–D** The expressions of E-cad, N-cad, Vimentin, and Slug were detected by Western blot. **C–E** The protein ratios in TU686, TU686/CAF + IL-33Ab, TU212, TU212/CAF + IL-33Ab, TU686/CAF + IgG, and TU212/CAF + IgG cells (*p < 0.05)

EMT, as a key event in tumor cell migration, invasion, and metastasis, is linked to strong interactions between the cancer microenvironment and cancer cells [5]. To explore the correlation between EMT and the metastasis of LSCC, we used immunofluorescence and Western blot assays to examine the expression levels of EMT-related proteins. As shown in Fig. 2A, the expression of vimentin was markedly increased, while the expression of E-cadherin was significantly decreased in TU686 cells co-cultured with CAFs treated with IL-33 antibody (TU686/CAF + IL-33Ab cells) compared to TU686 cells co-cultured with CAFs treated with IgG (TU686/CAF + IgG cells, p < 0.05). Moreover, as depicted in Fig. 2B–D, the expressions of N-cadherin, vimentin, and slug were markedly increased, while the expression of E-cadherin was significantly decreased in TU686/CAF + IL-33Ab and TU212/CAF + IL-33Ab cells compared to TU686/CAF + IgG and TU212/CAF + IgG cells (p < 0.05). The results of these experimental data indicate that IL-33 promoted the EMT of LSCC cells.

### CAF-derived IL-33 accelerated LSCC cells ability of movement, migration and invasion

**Fig. 3:**
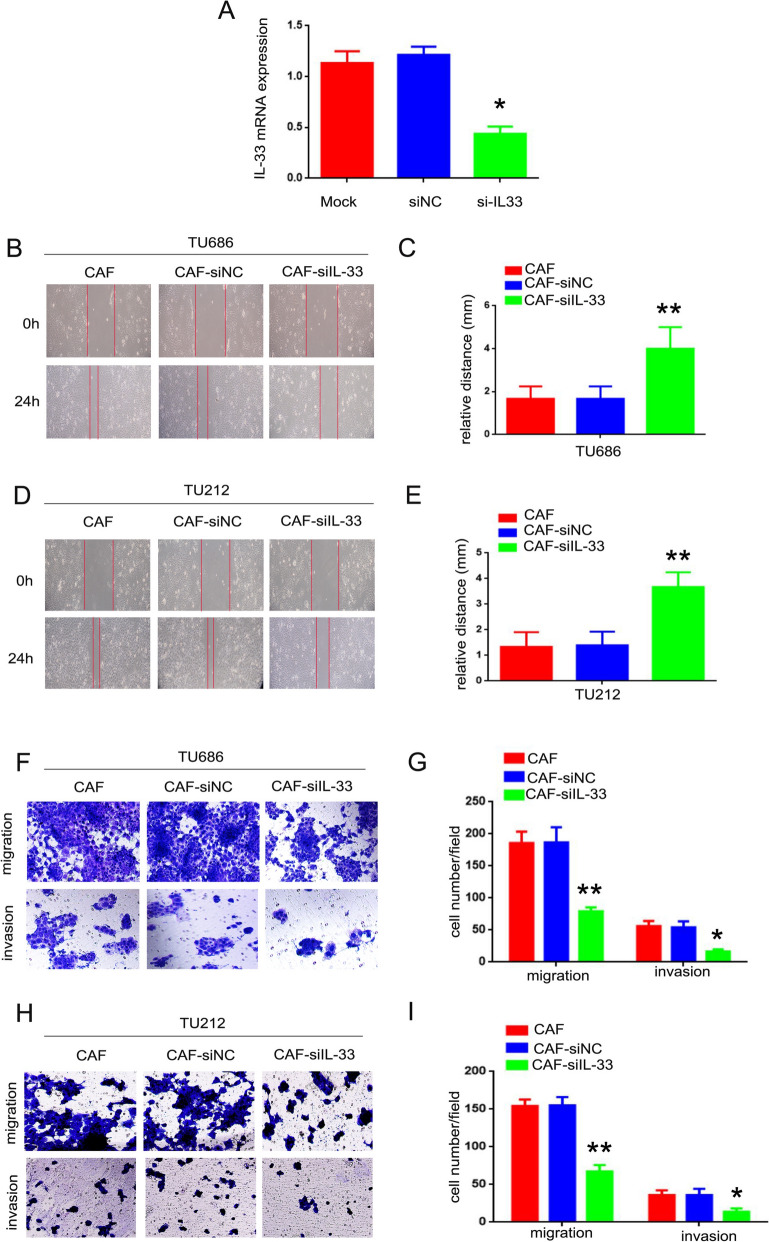
CAF-derived IL-33 accelerated LSCC cells’ abilities of movement, migration, and invasion. **A** The silencing effect of IL-33 (*p < 0.05). **B–D** The mobility of TU686/CAF-si-IL-33 and TU212/CAF-si-IL-33, TU686/CAF-siNC, and TU212/CAF-siNC cells was analyzed via wound-healing assay. **C–E** The relative distances of TU686/CAF, TU212/CAF, TU686/CAF-si-IL-33, and TU212/CAF-si-IL-33, TU686/CAF-siNC, and TU212/CAF-siNC cells (**p < 0.01). **F–H** The migration and invasion abilities of TU686/CAF, TU212/CAF, TU686/CAF-si-IL-33, and TU212/CAF-si-IL-33, TU686/CAF-siNC, and TU212/CAF-siNC cells were analyzed by Transwell assay. **G–I** The cell numbers in each field of TU686/CAF, TU212/CAF, TU686/CAF-si-IL-33, and TU212/CAF-si-IL-33, TU686/CAF-siNC, and TU212/CAF-siNC cells (*p < 0.05, **p < 0.01)

Inflammatory factors can promote the initiation and progression of tumors through a variety of mechanisms. To investigate whether IL-33 derived from CAFs promotes the metastasis of LSCC in vitro, CAFs were transiently transfected with IL-33 siRNA (si-IL-33) or a control scrambled siRNA (si-NC). The knockdown efficiency is shown in Fig. 3A (p < 0.05). And TU686 and TU212 cells co-cultured with CAFs transfected with si-IL-33 (TU686/CAF-si-IL-33 and TU212/CAF-si-IL-33 cells) exhibited reduced mobility compared to those co-cultured with CAFs transfected with si-NC (TU686/CAF-si-NC and TU212/CAF-si-NC cells). This difference was statistically significant (Fig. 3B–E, p < 0.01). Furthermore, the migration ability of TU686/CAF-si-IL-33 (79 ± 3.8) and TU212/CAF-si-IL-33 (67 ± 4.9) cells was reduced compared to TU686/CAF (185 ± 10.4), TU212/CAF (154 ± 4.9), TU686/CAF-si-NC (186 ± 13.7), and TU212/CAF-si-NC cells (155 ± 6.3, Fig. 3F–I, p < 0.01). Similarly, the invasion ability of TU686/CAF-si-IL-33 (16 ± 2.0) and TU212/CAF-si-IL-33 (13 ± 2.6) cells was reduced compared to TU686/CAF (56 ± 4.7), TU212/CAF (38 ± 3.6), TU686/CAF-si-NC (54 ± 5.2), and TU212/CAF-si-NC cells (38 ± 4.7, Fig. 3F–I, p < 0.05). The results of these experimental data indicate that IL-33 derived from CAFs accelerates the mobility, migration, and invasion of LSCC cells.

### CAF-derived IL-33 promoted the EMT of LSCC cells

**Fig. 4:**
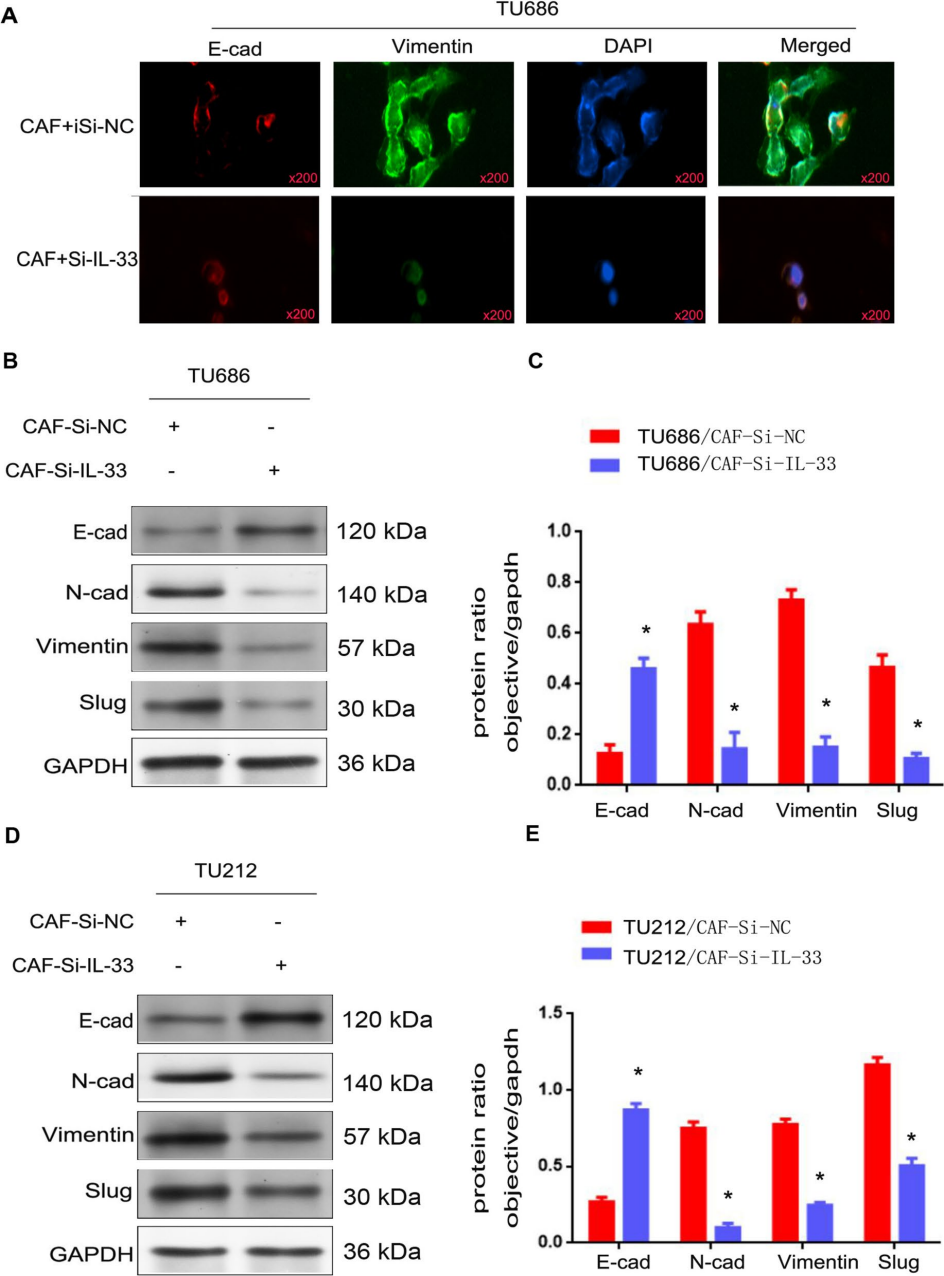
CAF-derived IL-33 promoted the EMT of LSCC cells. **A** The expressions of E-cad and Vimentin were detected by immunofluorescence assay in TU686/CAF-si-NC cells. **B–D** The expressions of E-cad, N-cad, Vimentin, and Slug were detected by Western blot in TU686/CAF-si-IL-33 and TU212/CAF-si-IL-33, TU686/CAF-siNC, and TU212/CAF-siNC cells. **C–E** The protein ratios in TU686/CAF-si-IL-33 and TU212/CAF-si-IL-33, TU686/CAF-siNC, and TU212/CAF-siNC cells (*p < 0.05)

To further investigate whether IL-33 derived from CAFs promotes the EMT of LSCC cells, immunofluorescence and Western blot assays were used to examine the expression levels of EMT-related proteins. As shown in Fig. 4A, the expression of vimentin was markedly increased, while the expression of E-cadherin was significantly decreased in TU686/CAF-siNC cells compared to TU686/CAF-si-IL-33 cells (p < 0.05). Moreover, as depicted in Fig. 4B–E, the expressions of N-cadherin, vimentin, and slug were markedly increased, while the expression of E-cadherin was significantly decreased in TU686/CAF, TU686/CAF-siNC, TU212/CAF, and TU212/CAF-siNC cells compared to TU686/CAF-si-IL-33 and TU212/CAF-si-IL-33 cells (p < 0.05). These observations indicate that IL-33 derived from CAFs promotes the EMT of LSCC cells.

### CAF-derived IL-33 promoted LSCC metastasis in vivo

**Fig. 5:**
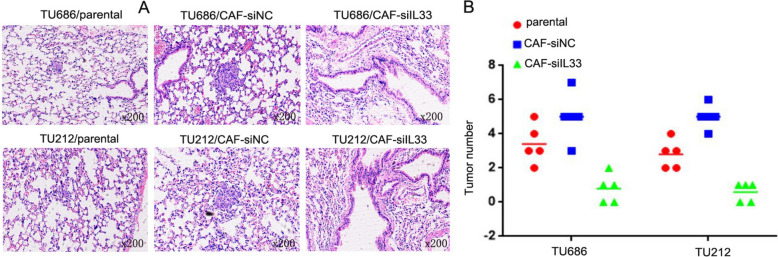
CAF-derived IL-33 promoted the metastasis of LSCC in vivo. **A** Pulmonary nodules were quantified by H&E staining. **B** Pulmonary tissue and nodules were quantified by H&E staining from TU686/CAF-si-IL-33, TU212/CAF-si-IL-33, TU686/CAF-siNC, and TU212/CAF-siNC cells (*p < 0.05)

To further clarify the mechanism of LSCC tumorigenesis and metastasis involving CAF-derived IL-33, TU686/CAF-si-IL-33 and TU212/CAF-si-IL-33 cells, along with TU686/CAF-siNC, TU212/CAF-siNC, T686/parental and TU212/parental cells, were inoculated into nude mice via tail vein injection. Four weeks post-inoculation, TU686/CAF-si-IL-33 (5 ± 0.88) and TU212/CAF-si-IL-33 cells (5 ± 0.57), which had lower IL-33 expression, demonstrated fewer and smaller lung metastases compared to TU686/parental (3.4 ± 1.14), TU212/parental (2.8 ± 0.84), TU686/CAF-siNC (0.8 ± 0.84) and TU212/CAF-siNC cells (0.6 ± 0.55) with higher IL-33 expression (Fig. 5A–B, p < 0.05). These results suggest that IL-33 derived from CAFs can promote the metastasis of LSCC in vivo.

## Discussion

Laryngeal squamous cell carcinoma (LSCC) is a malignant tumor derived from the mucosal epithelial tissue of the larynx. Despite standard radical surgery, the prognosis for LSCC patients remains poor, primarily due to metastasis. Therefore, it is imperative to clarify the specific molecular mechanisms involved and identify new therapeutic targets to develop more effective prevention and treatment strategies for clinical practice.

Epithelial-mesenchymal transition (EMT), a key event in tumor cell invasion and metastasis, is influenced not only by the tumor itself but also by the interaction between tumor cells and their microenvironment. This microenvironment comprises a complex integrated system of tumor cells, host cells, and matrix components [6]. Currently, it is believed that the unique cell–matrix changes within the tumor microenvironment not only accompany the malignant transformation of normal epithelial cells but also promote tumor cell metastasis [7]. Additionally, the metastasis of different tumors exhibits organ specificity, meaning they have a predilection for different target organs. It is generally accepted that the tumor microenvironment influences the distribution and movement of malignant tumors, and the development of metastatic cancer in secondary organs results from interactions between disseminated tumor cells and site-specific microenvironments. Recent studies have suggested that tumor stromal fibroblasts do not merely play a passive supportive role but also act as potential tumor promoters. However, the specific molecular mechanisms by which tumor stromal fibroblasts contribute to tumor development and the regulatory mechanisms governing their function remain unclear. Thus, in-depth study of cancer-associated fibroblasts (CAFs) is crucial for elucidating the molecular mechanisms of tumorigenesis and progression, providing new theoretical foundations and intervention strategies for early tumor diagnosis. Previous studies have shown that CAFs significantly promote the metastasis of LSCC [10]. However, the specific molecular mechanisms underlying CAF activation in the context of EMT in LSCC have not been extensively studied. Further exploration of these mechanisms is essential.

In the realm of tumor metastasis, extensive research has demonstrated that tumor genesis initially involves a process of injury and chronic inflammation, which recruits various inflammatory cells and bone marrow mesenchymal stem cells, creating an optimal environment for subsequent colony formation and metastasis. This is followed by stromal reorganization to generate a more conducive environment for proliferation, ultimately leading to metastasis. All results from our study indicate that IL-33 promoted the migration, invasion, and EMT of LSCC cells. In the co-culture model of LSCC cells and CAFs, the use of a neutralizing antibody to silence IL-33 expression inhibited the migration, invasion, EMT, and metastasis potential of LSCC cells both in vitro and in vivo. In conclusion, IL-33 derived from CAFs mediates EMT to promote the metastasis of LSCC cells.

Previous studies have shown that inflammation is a complex defense mechanism of living tissues with a vascular system against harmful agents. During the processes of inflammation and carcinogenesis, inflammatory factors can promote the initiation and progression of tumors through various mechanisms. IL-33 is a recently identified cytokine and a new member of the interleukin-1 family. Earlier studies on IL-33 primarily focused on allergic reactions, inflammation, infections, autoimmune diseases, heart and lung diseases, among others. Recently, abnormal expression of IL-33 has been noted in the progression of numerous cancers, and the related signaling pathway has become a convergence point for many oncogenic signals, playing a pivotal role in tumor development and progression. Overexpression of IL-33 has been associated with chemotherapy resistance, migration, and metastasis in various cancers [11], and IL-33 can promote lung tumorigenesis and metastasis via type 2 innate lymphoid cells (ILC2s) [12]. IL-33 can also stimulate the production of pro-inflammatory cytokine IL-8, potentially contributing to inflammation-related pancreatic cancer [13]. Furthermore, IL-33 levels are significantly elevated in head and neck squamous cell carcinomas (HNSCC) and are closely associated with the depth of invasion and distant metastasis of HNSCC [14].

This study still has certain limitations. We did not directly isolate cancer-associated fibroblasts (CAFs) from mouse lung tissues to verify the differences in IL-33 expression between groups, although the consistent phenotypic changes observed between our control and experimental groups provide strong support for the role of IL-33 in promoting tumor progression within the tumor microenvironment. Additionally, while the importance of detecting receptor ST2 and its downstream regulators (such as the ERK pathway and ZEB2) is recognized, these specific molecules were not analyzed by Western blot due to the focus of this study on establishing the foundational role of IL-33. Future studies should further explore these downstream signaling pathways to comprehensively elucidate their mechanisms and refine our understanding of the functional role of CAF-derived IL-33 in tumor development.

While in vitro and in vivo co-culture models were employed to demonstrate the effects of IL-33 on LSCC progression, the use of xenograft models or genetically engineered mouse models could provide more physiological relevance and a deeper understanding of tumor-stroma interactions in a whole-organism context. The current model might not entirely replicate the intricate microenvironment found in human cancers. Although the study identifies IL-33 as a mediator of EMT and metastasis in LSCC, the detailed molecular mechanisms by which IL-33 influences CAF activation and subsequent tumor behavior remain partially understood. Further investigation into the signaling pathways downstream of IL-33 and the crosstalk between CAFs and LSCC cells is warranted. The findings are based on experimental models and need validation in clinical settings. The extent to which the observed phenomena apply to human patients with LSCC requires additional epidemiological studies and clinical trials. It is also necessary to explore the potential of targeting IL-33 or its receptors as therapeutic strategies in a clinical context.

In summary, the findings of this research not only provide a new mechanism for the activation of CAFs and the metastasis of LSCC but also offer theoretical significance and application value for developing more effective prevention and treatment strategies in clinical practice.

## Materials and methods

### Cell culture

The LSCC cell lines (TU686 and TU212 cells) were obtained from the Shanghai Institutes for Biological Sciences, Chinese Academy of Sciences. The cells were maintained in MDEM medium supplemented with 10% fetal bovine serum (FBS) and 100 U/ml penicillin/streptomycin, purchased from YOBIBIO (Shanghai, China). Cultures were kept in a humidified atmosphere with 5% CO2 at 37 °C. Tumor adjacent non-tumor tissues were mechanically minced into small pieces (1–1.5 mm^3^) and seeded onto 10 cm petri dishes in DMEM with 10% FBS containing 100 IU/ml penicillin and 100 IU/ml streptomycin. A homogeneous group of fibroblasts, produced after 7–14 days of culture, would be used in the experiments.

### Wound healing assay

Cells were seeded at 1 × 10^6 cells/well in six-well plates until confluent. A scratch was made with a 200 μl pipette tip, and the medium was replaced with fresh medium containing 1% FBS. Photographic images were taken at 0 and 24 h post-scratch.

### Immunofluorescence

Cells were fixed with 4% paraformaldehyde for 30 min, then rinsed with phosphate-buffered saline (PBS) for three times, blocked with 5% BSA for 1 h at 37˚C. They were incubated with the PathScan EMT Duplex IF Kit (Cell Signaling Technology, Boston, MA, USA) and subsequently blocked with normal non-immune goat serum for 30 min at 37 °C. After three washes with PBS, cells were stained with Alexa Fluor-conjugated secondary antibodies (1:1000; cat. no. 4413S; Cell Signaling Technology, Inc.) and DAPI (1:1000; Beyotime Institute of Biotechnology).

### Western blot

Detailed procedures for Western blotting are described in our previous publication [6]. Antibodies against P130 (1:2000, #13,383), E2F4 (1:2000, #40,291), E-cadherin (1:2000, #14,472), N-cadherin (1:2000, #14,215), Vimentin (1:2000, #5741), and Slug (1:2000, #9585) were obtained from Cell Signaling Technology (CST). Membranes were visualized using Thermo Pierce chemiluminescent (ECL) Western Blotting Substrate (Thermo Fisher Scientific, Waltham, MA, USA) with a Tanon 5200 system (Tanon, Shanghai, China).

### Migration and invasion assays

For the migration assay, 2 × 10^5 cells suspended in 200 μL of serum-free DMEM were placed in the upper chamber of a Transwell insert (Corning Inc., Corning, NY). The lower chamber contained 600 μL of DMEM supplemented with 10% FBS. After 24 h, cells that had migrated to the lower surface were fixed with 4% paraformaldehyde, stained with crystal violet solution, and imaged under a microscope at 10 × magnification.

For the invasion assay, the upper chamber was coated with Matrigel (BD Labware, Bedford, MA) before seeding cells. The rest of the procedure was the same as the migration assay.

### Animal experiments

Animals were sourced from the Institute of Zoology, Chinese Academy of Sciences. Animals were approved by based on the protocol was approved by the Experimental Animal Ethics Committee at Shanghai Jiao Tong University School of Medicine in accordance with the Institutional Animal Care and Use Committee. At the conclusion of the experiments, euthanasia was performed via intraperitoneal injection of an overdose of sodium pentobarbital (4%, 200 mg/kg; Sigma, Shanghai, China). At the end of the experimental period, animals were euthanized under isoflurane anesthesia, and lung samples were subsequently collected from each group for further analysis.

### H&E staining methodology

Formalin-fixed, paraffin-embedded tissue Sects. (5 μm thickness) were deparaffinized in xylene and rehydrated through graded ethanol solutions. Sections were then stained with hematoxylin for 5 min, washed in running tap water, differentiated in acid alcohol, and counterstained with eosin Y solution for 1 min. After dehydration and clearing, sections were mounted with DPX mounting medium and visualized under a light microscope (Olympus Corporation).

### Statistical analysis

Data are presented as mean ± SD. Significant differences between groups were determined using Student's t-test and one-way ANOVA (GraphPad Prism software v6.0). A p-value < 0.05 was considered statistically significant.

## Supplementary Information

Supplementary material 1.

## Data Availability

No datasets were generated or analysed during the current study.
