# Supplementary figures and images for: CAF derived IL-33 mediated EMT to promote the metastasis of LSCC cells
Source: Eur J Med Res. 2025 Nov 21;30:1147. doi: 10.1186/s40001-025-03431-4 (PMC12636156; doi:10.1186/s40001-025-03431-4)

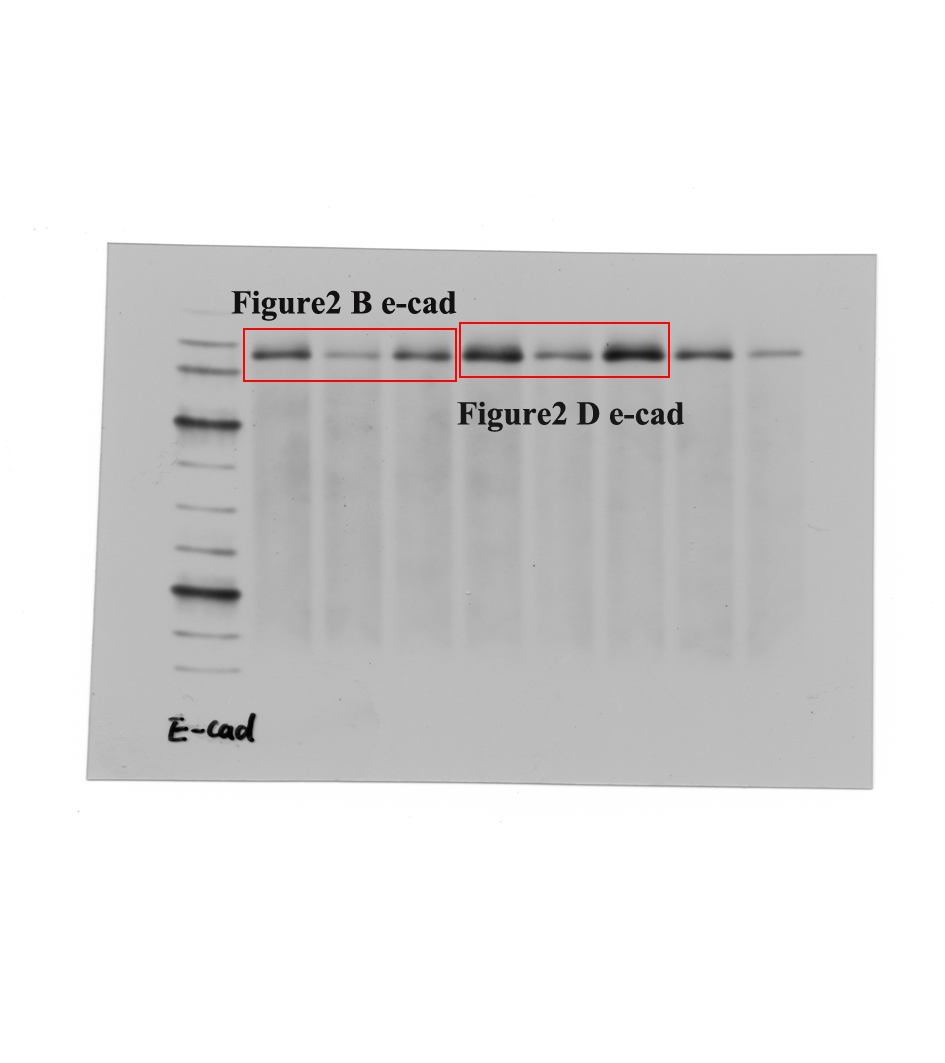


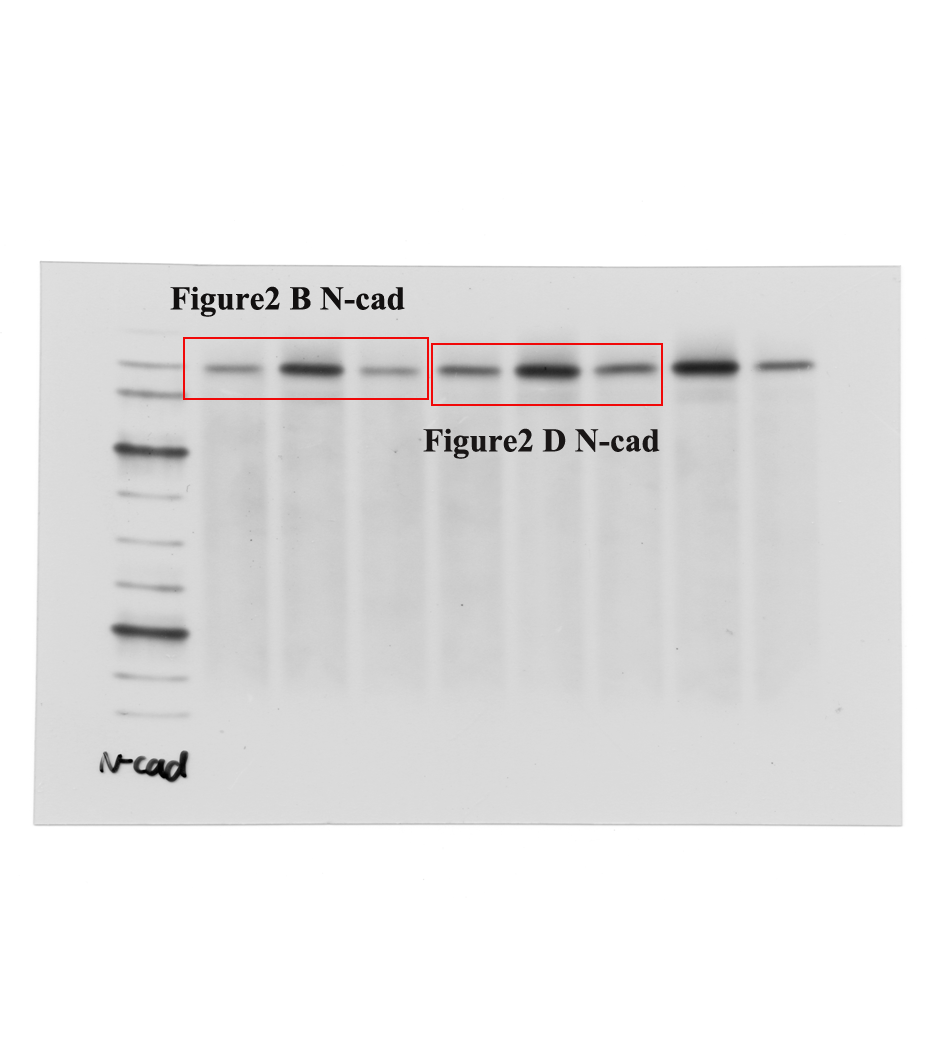


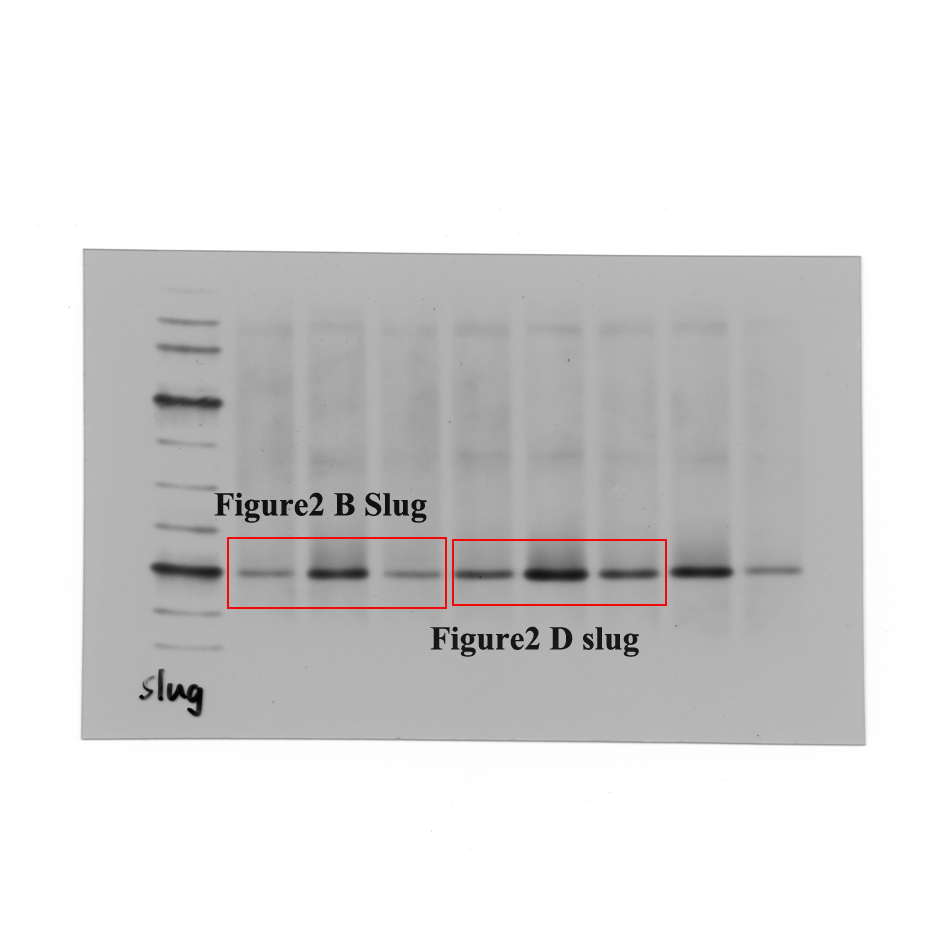


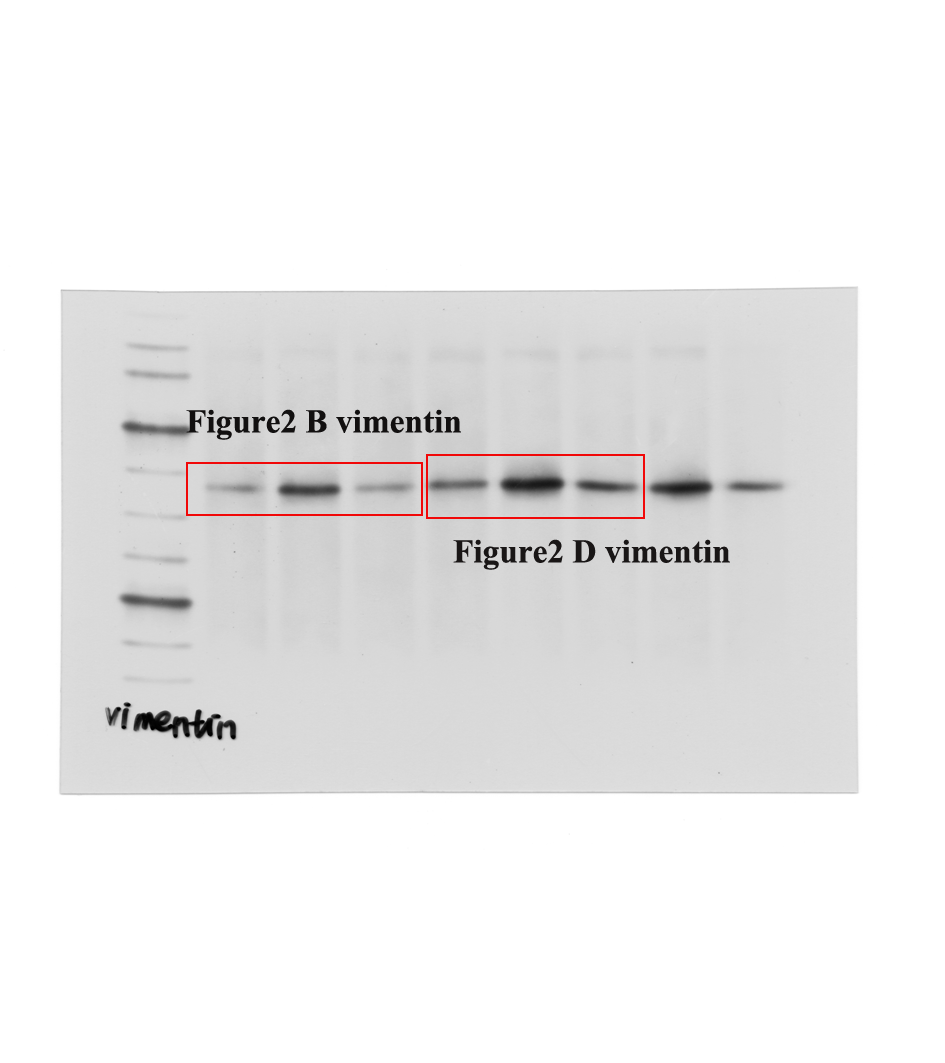


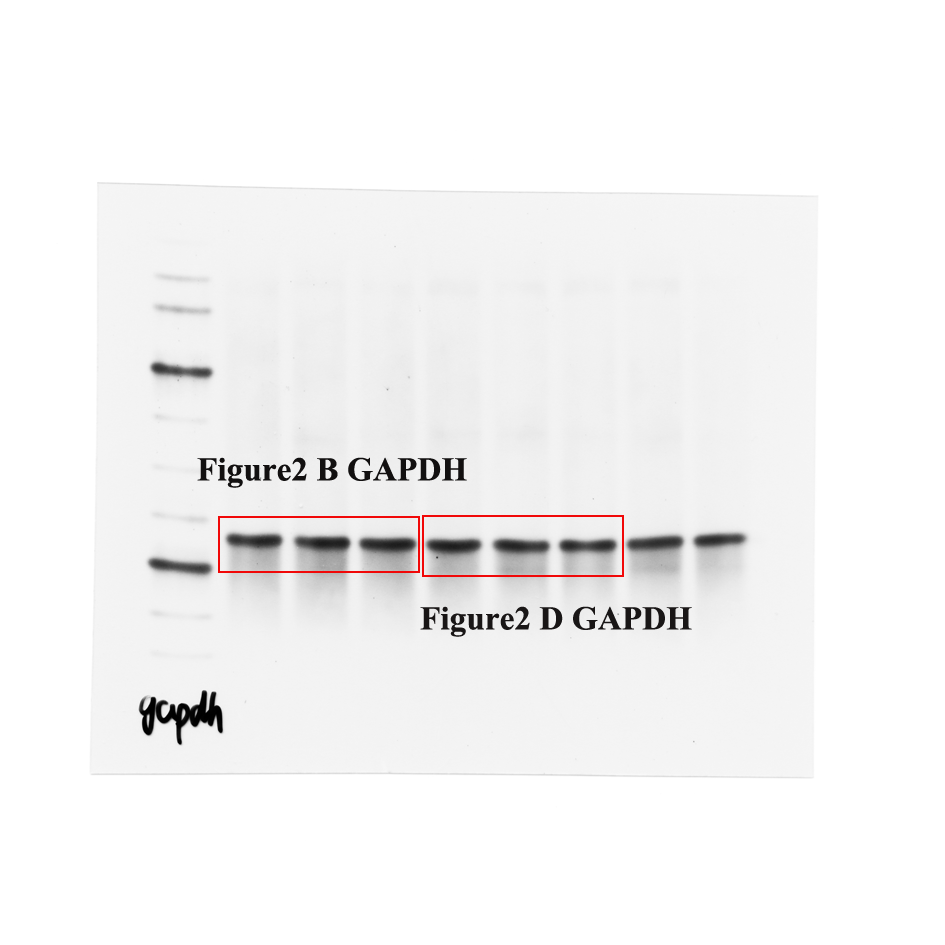


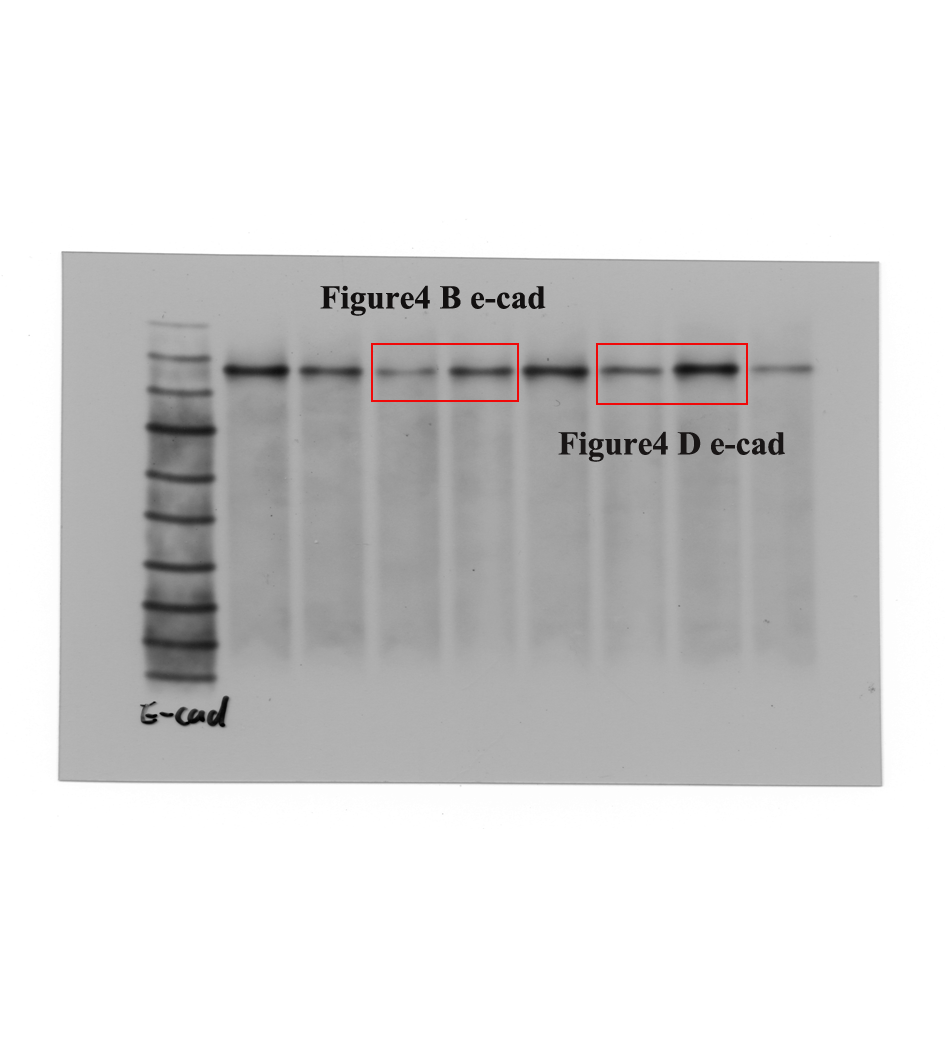


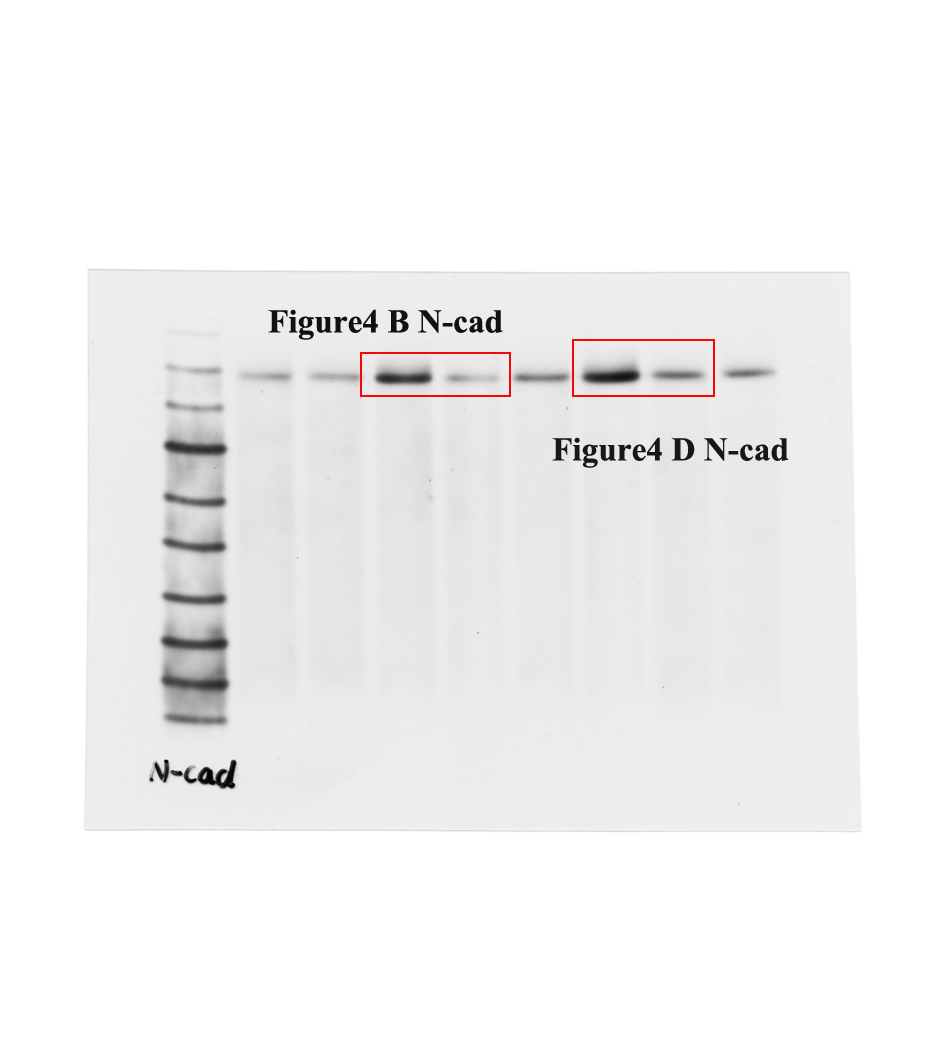


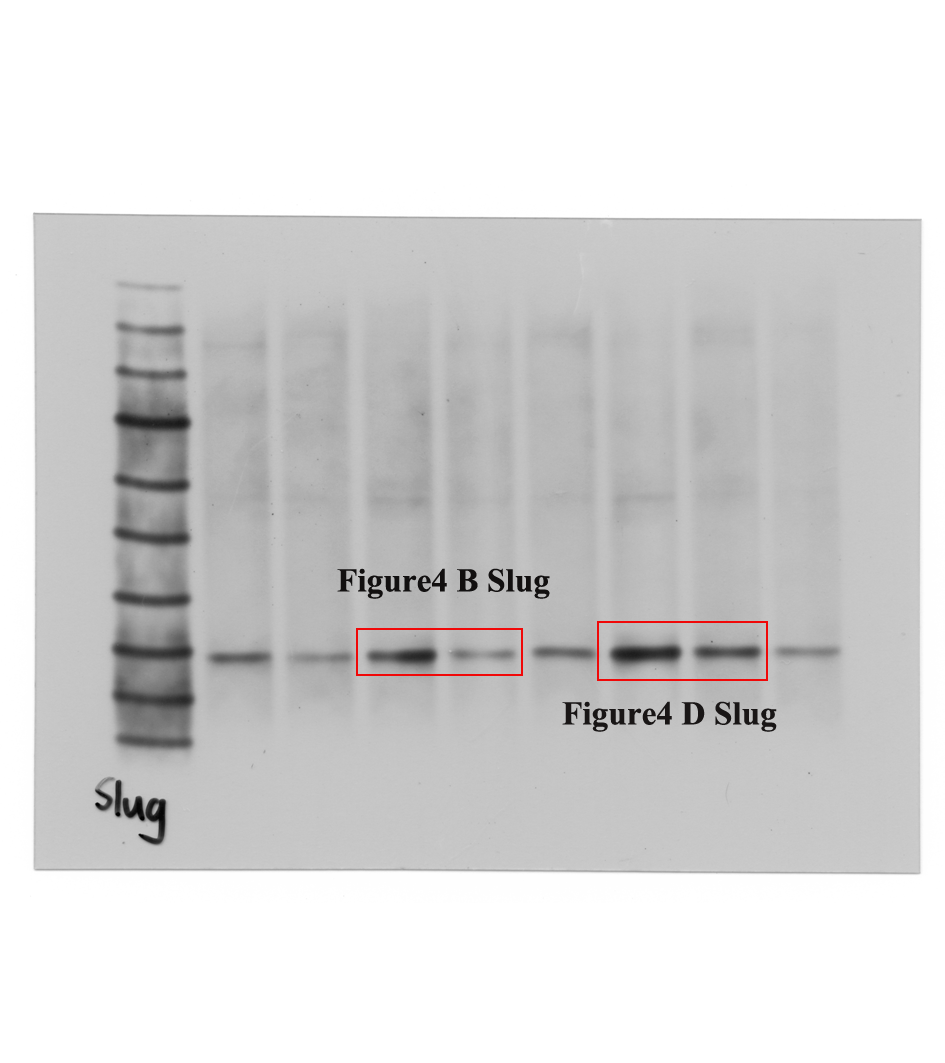


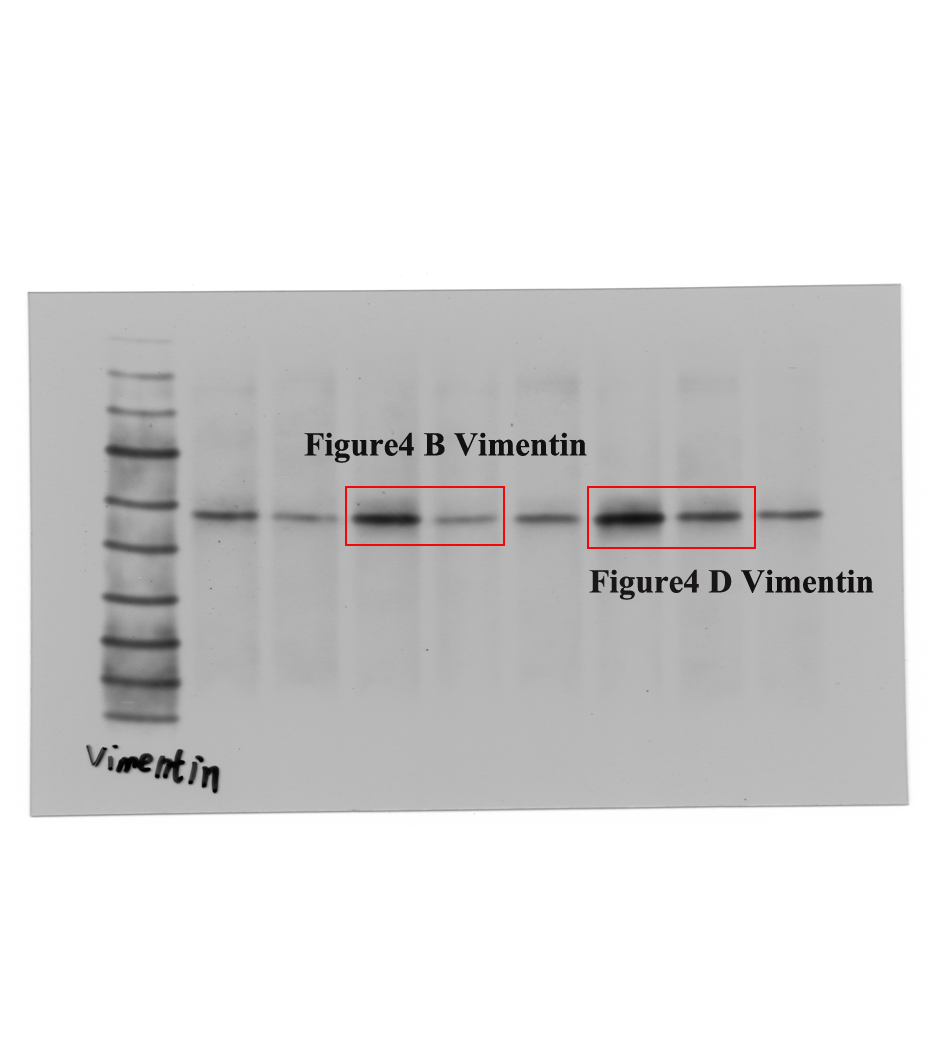


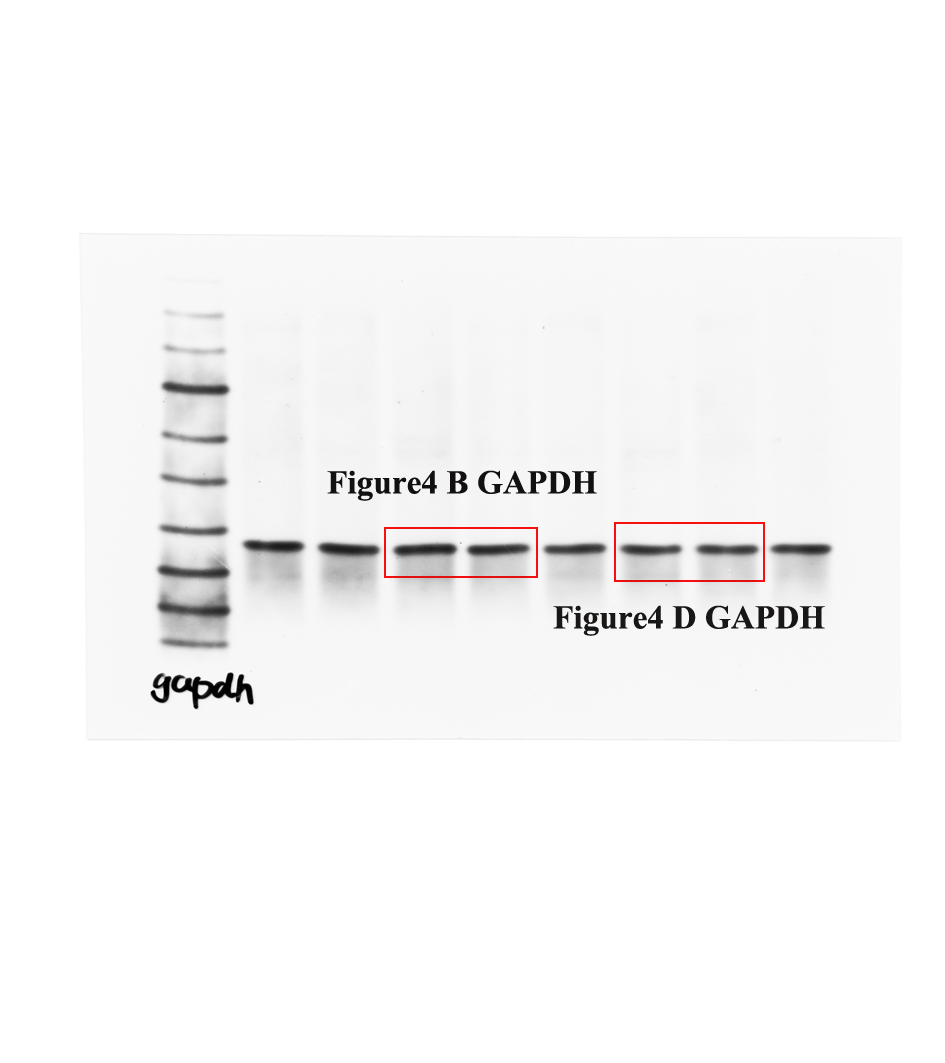

Supplement: Supplementary file 1 — Supplementary material 1. [file 40001_2025_3431_MOESM1_ESM.docx]
